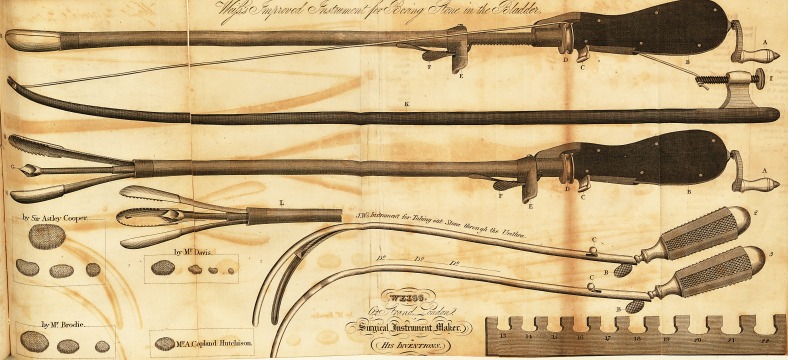# Intelligence, &c.

**Published:** 1825-01-01

**Authors:** 


### XII.
### INTELLIGENCE, &c.

### 1. LITHOTOMY.

J. Weiss respectfully submits to the inspection of Professional Gentlemen, and of the Public in general, an Instrument capable of being passed through the urethra, and adapted for perforating and breaking the stone in the bladder. He takes this occasion to state the reasons which urged him to accomplish this important desideratum in surgery. Frequent mention had been made, in the newspapers and other journals, of an instrument of this description constructed by Dr. Civiale of Paris, to whom several eminent surgeons in this country had written, requesting to be supplied with it, but without success, as he did not wish it to become public. Various applications were, in consequence, made to J. Weiss; and his answer was, that if any person could introduce a straight instrument into the bladder through the urethra, he could invent such an instrument. He instantly proceeded to make the attempt; and its result has justified his expectations. The instrument constructed by him has been approved by competent judges, and has been pronounced, by a French surgeon, who has examined it, to be of much smaller dimensions, and more simple in its construction, than that of Dr. Civiale. Having taken neither that nor any other for his model, J. Weiss considers himself warranted in calling the present invention his own; but he leaves the question of its merits to be decided by public opinion.

He has thought proper to annex to the engraving of it, a representation of his Instrument for extracting Calculi through the Urethra, which has been used with very great success. Its claims to preference he submits to the candid and equitable judgment of the Profession.

---

### 2. NATURAL HISTORY, BOTANY, &c.

The whole, or any part of the following Books, may be bought for one third less than their original prices. They are all in excellent order, and may be seen any morning before 1 o'clock, at Mr. Soles', 7, *Gray's Inn Square*.

Hill's, Sir John, Vegetable System, vols. 26 in 13, large folio, ½ bound, ed. 1773.—Hill's British Herbal, vol. 1, large folio, bound, plates colored, edition 1756.—Merions Surinam Insects, Fruits & Flowers, vol. 1, large folio, bd. English MS. translation from the original, plates cold. ed. 1719.—Curtis's Flora Londinensis, vols. 6, large folio, bd. plates cold. ed. 1777.—Philosophical Transactions abridged, vols. 18, 4to. handsomely ½ bd. & lettered, ed. 1809.—Sydenham's, Edwards' Botanic Garden, vols. 2, large 4to. well bd. plates beautifully colored, ed. 1812.—Dictionnaire Raisonné d'Histoire Naturelle, vols. 4, bd. 4to. ed. 1768.—Thornton's Elements of Botany, vols. 2, large 8vo. neatly bd. ed. 1812.—Donovan's

British Insects, vols. 10 in 5, large 8vo. handsomely bd. plates beatifully col'd. ed. 1800.—Linnæus' System of Nature, translated by Turton, vols. 4. large 8vo. well bd. ed. 1800.—Linnæus' Genera Vermium, translated by Basbert, vol. 1, 4to. handsomely bd. beautifully cold. ed. 1788.—Les Genus des Inscites, translated by Basbert, vol. 1, 4to. bd. Ellis's Zoophytes, by Solander, vol. 1, handsomely bd. fine paper, ed. 1786.—Ellis's Coralines, vol. 1, 4to. bd. handsomely, fine paper, ed. 1755.—Rashleigh's Cornish Minerals, vol. 1, 4to. handsomely bd. beautifully col'd. fine paper, ed. 1797.—Woodville's Medical Botany, vols. 4, 4to. handsomely bd. col'd. plates, ed. 1810.—Pennant's British Zoology, vols. 4, 8vo. handsomely bd. col'd. ed. 1776.—Ellis's Manjostan, and Langhorn's Fables of Flora, bd. together in 1 vol.—Bryant's Flora Dietetica, vol. 1, 8vo. bd. ed. 1783.—Lees' Introduction to Botany, vol. 1, 8vo. ½ bd. ed. 1794.—Smith's English Flora, vol. 2, bd. ed. 1824.—Donn's Hortus Cantabrigiensus, vol. 1, bd.—Conversations on Botany, vol. 1, 8vo. bds. plates cold. ed. 1820.—Spallanzani on the Natural History of Animals and Vegetables, vols. 2, well bd. large 8vo. edition 1803.

---

3. The Plan of Lectures on the Anatomy, Physiology, and Pathology of the Ear, by JOHN HARRISON CURTIS, Esq. F.M.S. Aurist to His Majesty, and their Royal Highnesses the Duke and Duchess of Gloucester, Surgeon to the Royal Dispensary for Diseases of the Ear, &c. to commence on *Thursday, January 5th*, 1825, at Seven o'Clock in the Evening, at the Royal Dispensary for curing Diseases of the Ear, 10, *Dean Street, Soho Square.*

*Arrangement of the Course.*—In the Introductory Part, will be considered the Importance of the Sense of Hearing as the medium of social intercourse; the various degrees of this Sense in the several tribes of Animals; with the different construction of the Organ for that purpose.

The Anatomy of the Human Ear will be described, as divided into external, intermediate, and internal parts; and the description will be illustrated by Anatomical Preparations.

The Physiology, or Uses of the different parts of the Ear, will next be examined.

On finishing the Structure and Uses of the Ear, the various Diseases occasioning Deafness will then be considered, treating them in the same order in which the Structure has been described.

This order will comprehend, first, the Affections of the Meatus Auditorius, or external ear; 2ndly, those of the Tympanum, *viz.* its puriform discharge, and the Obstruction of the Eustachian Tube, with the Operation; 3rdly, the Diseases of the Labyrinth, whether constitutional, as nervous, scrofulous, syphilitic, &c.; or local, as Paralysis of the Auditory Nerve, defective Organization, &c.

The Subject will conclude with general Remarks, applicable to this class of Diseases; to which will be added, Considerations on the best means of treating the Deaf and Dumb.

A CLINICAL LECTURE will be given occasionally on the most important Cases that occur at the Royal Dispensary.

The Royal Dispensary is open to Pupils:—TERMS.—*Attendance:* Three Months, Five Guineas; Six Months, Eight Guineas; Perpetual, Ten Guineas. *Lectures:* Single Course, Two Guineas; Two Courses, Three Guineas; Perpetual, Five Guineas.

*For Particulars, apply to* Mr. CURTIS, *at his House, No. 2, Soho Square.*

---

4. A complete Edition of the Works of the late Dr. BAILLIE, with an Account of his Life, collected from the most authentic sources, will speedily be published by Mr. WARDROP.

## Figures and Tables

**Figure f1:**